# Trends, lag and characteristics of rare disease drug approval in the USA and China, 1983–2022

**DOI:** 10.1186/s13023-025-04175-4

**Published:** 2025-12-22

**Authors:** Shaohong Wang, Xin Liu, Yuzhen Zou, Yan Tang, Wei Zuo, Rong Jiang, Junmei Shang, Xin Tian, Qingyang Liu, Tingting Xu, Bo Zhang, Shuyang Zhang

**Affiliations:** 1 Department of Pharmacy, Peking Union Medical College Hospital (Dongdan campus), Chinese Academy of Medical Sciences, No.1 Shuaifuyuan Wangfujing Dongcheng District, Beijing, 100730 China; 2 State Key Laboratory of Complex Severe and Rare Diseases, Peking Union Medical College Hospital, Chinese Academy of Medical Sciences, Beijing, 100730 China; 3 Department of Cardiology, Peking Union Medical College Hospital, Chinese Academy of Medical Sciences, Beijing, 100730 China; 4 National Center for Healthcare Quality Management in Rare Diseases, Beijing, 100730 China; 5 China Pharmaceutical University, Nanjing, 211198 China

**Keywords:** Pharmaceutical management and regulations, Orphan drug, Rare disease, Pharmacy, Accessibility, Availability

## Abstract

**Background:**

Over the past approximately 40 years, Chinese drug regulations have undergone many major reforms to accelerate the approval of drugs and keep pace with the scientific innovation of drugs in the world, especially developed countries. In 2018 and 2023 China’s National Health Commission in collaboration with other departments released China’s “First Batch of Rare Diseases Catalogue” and “Second Batch of Rare Disease Catalogue”. However, there is currently less relevant research on the overview and speed of the approval of rare disease drugs in China.

**Methods:**

This study used cross-sectional analysis of rare disease drugs approved in China and the USA from 1983 to 2022 through official drug search databases and systematically analyzed and compared rare disease drugs approved in the USA and China, including the number, approval time, related laws and regulations, and expedited programs.

**Results:**

Between 1983 and 2022, the USA Food and Drug Administration (FDA) approved a total of 693 orphan drugs (covering 1228 formulations/specifications). Among these, 201 drugs (425 formulations/specifications) were approved by China’s National Medical Products Administration (NMPA), accounting for 29.0% (201/693) of the approved drugs and 34.6%(425/1228) of the approved formulations/specifications. The number of China’s rare diseases drugs is increasing year by year, and the approval speed has gradually accelerated. The median approval lag time was 32.3 years (IQR: 22.9–33.6) from 1983 to 1987. In contrast, the shortest median lag time occurred between 2018 and 2022, at approximately 1.4 years (IQR: 0.5–2.6)—a reduction of 94.2% that reflects the success of China’s ongoing drug regulatory reforms. The special procedures for drug approval have a great promoting effect on rare diseases drugs’ approval.

**Conclusion:**

This research provides evidence of breakthroughs in the review and approval of rare disease drugs in China and demonstrates the tremendous boost to rare disease drugs from China’s ongoing restructuring and reform of the drug regulatory ecosystem, as well as a stimulus for future rare disease drugs development in China.

**Supplementary Information:**

The online version contains supplementary material available at 10.1186/s13023-025-04175-4.

## Background

Orphan drugs, also known as rare disease drugs, are pharmaceuticals used for the treatment, prevention, and diagnosis of rare diseases [1]. Divergence exist in the defining criteria for rare diseases among different countries [2, 3]. The United States defines a rare disease as one affecting fewer than 200,000 individuals annually [4]; Japan classifies a disease as rare if it affects fewer than 50,000 patients or has a prevalence rate below 1 in 2,500 [5, 6]; In the European Union, the threshold is set at no more than 5 cases per 10,000 people [7]; In contrast, China currently adopts a catalog-based approach to define rare diseases. The First Catalogue of Rare Diseases was issued on May 11, 2018, encompassing 121 conditions [8]. This was followed by the Second Catalogue on September 18, 2023, which added 86 rare diseases [9]. It is expected that this catalog will be periodically updated to include more diseases in the future. Over the past five years, the development of orphan drugs in China has accelerated markedly, with the number of drugs in the pipeline increasing at an average annual rate of 34%—approximately 42% higher than the global growth rate [10]. While China has established official catalogs for defining rare diseases, it has not yet published a national list of officially designated orphan drugs. Therefore, this study analyzes the availability of USA approved orphan drugs in China and examines the differences in approval time. The USA established its legal framework for rare diseases earlier than China, and the regulatory systems for designating orphan drugs differ significantly between the two countries. Special FDA designations shorten clinical development and FDA approval times for new drugs treating rare and severe diseases with unmet medical needs [11]. Thus, this study addresses two primary questions: (1) Are orphan drugs approved in the USA also approved in China? (2) If so, what is the lag time for approval in China? By investigating these questions, we aim to elucidate the progress in the regulatory framework for rare disease drugs in China over a 40-year period (1983–2022).

Historically, drug approval lag in China was a significant issue [12, 13], primarily attributed to severe application backlogs, prolonged regulatory review times, and an inefficient clinical trial approval system. In response, China initiated a series of drug regulatory reforms starting in 2015, with the primary goal of establishing an effective review process by eliminating the backlog of overdue applications [14], Subsequent policy measures included expanding the reviewer team, implementing a 60-day default approval period for Investigational New Drug (IND) applications, introducing accelerated approval pathways, permitting simultaneous global Phase I clinical trials for imported drugs, and accepting multiregional clinical trial (MRCT) data for approval in China [15]. These efforts reflect China’s progress toward a more standardized and comprehensive ecosystem for rare diseases [15–18]. As a result of these reforms, since 2019, new drug applications can now be approved within the statutory timeline, with fast-track channels reducing approval delays by approximately 30 months. Although clinical trial applications have increased significantly since 2016, detailed analysis of approval delays remains limited [14]. Moreover, the immediate positive effects of the reforms may have overshadowed the persistent underlying causes of drug approval lags. This study aims to investigate the approval status in China of orphan drugs designated and approved by the USA FDA between 1983 and 2022. We analyze the extent of approval delays, compare special regulatory pathways in the USA and China, and provide an overview of the current orphan drug approval landscape. By mapping these patterns, our findings highlight the tangible achievements of China’s drug reform policies and offer empirical evidence to inform Chinese pharmaceutical companies, regulatory agencies, and drug R&D departments.

## Methods

### Data source and collection

In 2018, China’s National Health Commission issued the Working Procedures for Developing a Catalogue of Rare Diseases. This document stipulates that the catalogue will be updated periodically and that included diseases must simultaneously satisfy four conditions: (i) evidence of low incidence or prevalence, both internationally and domestically; (ii) severe impact on the health of patients and their families; (iii) the availability of a clear diagnostic method; and (iv) the existence of a treatment or intervention that is economically accessible, or alternatively, inclusion in a national research program if no effective treatment exists. To date, China has released two batches of its Rare Disease Catalogue, encompassing a total of 207 diseases, with future updates anticipated. Although the definitional approaches to rare diseases differ between China and the United States—and the USA recognizes a larger number of rare conditions—many diseases designated as rare in the USA are also present in the Chinese population. These patients equally require effective pharmacological treatment. Therefore, the primary focus of this article is on orphan drugs themselves, rather than on the diseases. This study specifically examines whether orphan drugs approved in the USA are also available in China and analyzes the differences in approval time between the two countries. Using publicly available data, we constructed a new database of orphan drugs approved in both the USA and China between January 1, 1983, and December 31, 2022. This database serves to summarize trends, approval speeds, characteristics, and outcomes of orphan drug approvals in China.

The primary data sources for this study included:

i. Drugs@FDA (https://www.fda.gov/drugs/development-approval-process-drugs/drug-approvals-and-databases).
ii. The FDA Orphan Drug Designation and Approval Online Database (https://www.accessdata.fda.gov/scripts/opdlisting/oopd/).
iii. The NMPA Drug Query Database (https://www.nmpa.gov.cn/datasearch/search-result.html).
iv. The NMPA Center for Drug Evaluation (CDE) website (https://www.cde.org.cn/main/xxgk/listpage/b40868b5e21c038a6aa8b4319d21b07d).

Data from these sources were integrated to construct a comprehensive database covering the period from January 1, 1983, to December 31, 2022. This database was designed to identify whether USA-approved orphan drugs were available in China, along with their corresponding approval dates and other relevant regulatory details. From the FDA databases, we extracted the following information for each orphan drug: non-proprietary name, trade name, orphan drug designation date, designation status, USA approval date, labeled indications, and expedited review programs (e.g., Priority Review, Fast Track, Breakthrough Therapy). Our analysis encompassed New Molecular Entities (NMEs) approved by the FDA’s Center for Drug Evaluation and Research (CDER), including chemical drugs approved via New Drug Applications (NDAs) and biologics approved via Biologics License Applications (BLAs).

The approval status of these USA orphan drugs in China was subsequently verified using the NMPA and CDE databases. When necessary, the third-party database Insight China Pharma Data (https://db.dxy.cn/v5/home) was consulted to supplement missing information on approved dosage forms, specifications, and initial approval dates, as official NMPA and CDE announcements occasionally lacked complete historical records.

### Data analysis

In this study, we assessed the relative drug approval lag by calculating the drug initial approval (DIA) lag, following the methodology of a previous study [19]. The DIA lag was defined as the time difference (in days) between the initial approval date of a rare disease drug in China and its initial approval date in the United States. A positive lag value indicates that the drug was approved in China later than in the USA, which was the most common scenario. Conversely, a negative value indicates earlier approval in China. The lag was calculated by subtracting the USA approval date from the Chinese approval date. The results were converted from days to years for graphical representation to enhance clarity. Drugs whose orphan drug designation was subsequently revoked by the FDA were still included in the analysis, as this study focuses on the timing of initial approvals. This project employs a cross-sectional analysis of drug approval data from 1983 to 2022.

## Results

### Trends

#### Number and trend of orphan drugs approved by the FDA and NMPA

Since the enactment of the Orphan Drug Act in 1983, the number of drug development programs receiving orphan drug designation and the number of FDA-approved orphan drug indications have increased annually. As of December 31, 2022, the FDA had granted 6,353 orphan drug designations, with an annual average of approximately 158.9.The number of designations remained relatively stable from 1983 to 2002, averaging 59.2 per year. However, from 2003 to 2022, designations increased steadily, reaching an annual average of 234.6 and peaking at 486 in 2017—approximately 12.5 times the number recorded in 1984.

Between January 1, 1983, and December 31, 2022, the FDA approved 1,108 orphan drug indications, covering 693 unique drugs. The annual number of approved indications also showed an upward trend, reaching a maximum of 95 in 2018. The proportion of new orphan drugs among all new drug approvals increased gradually over the 40-year period, reaching 46.3% (230 out of 497) from 2011 to 2022.

**Fig. 1:**
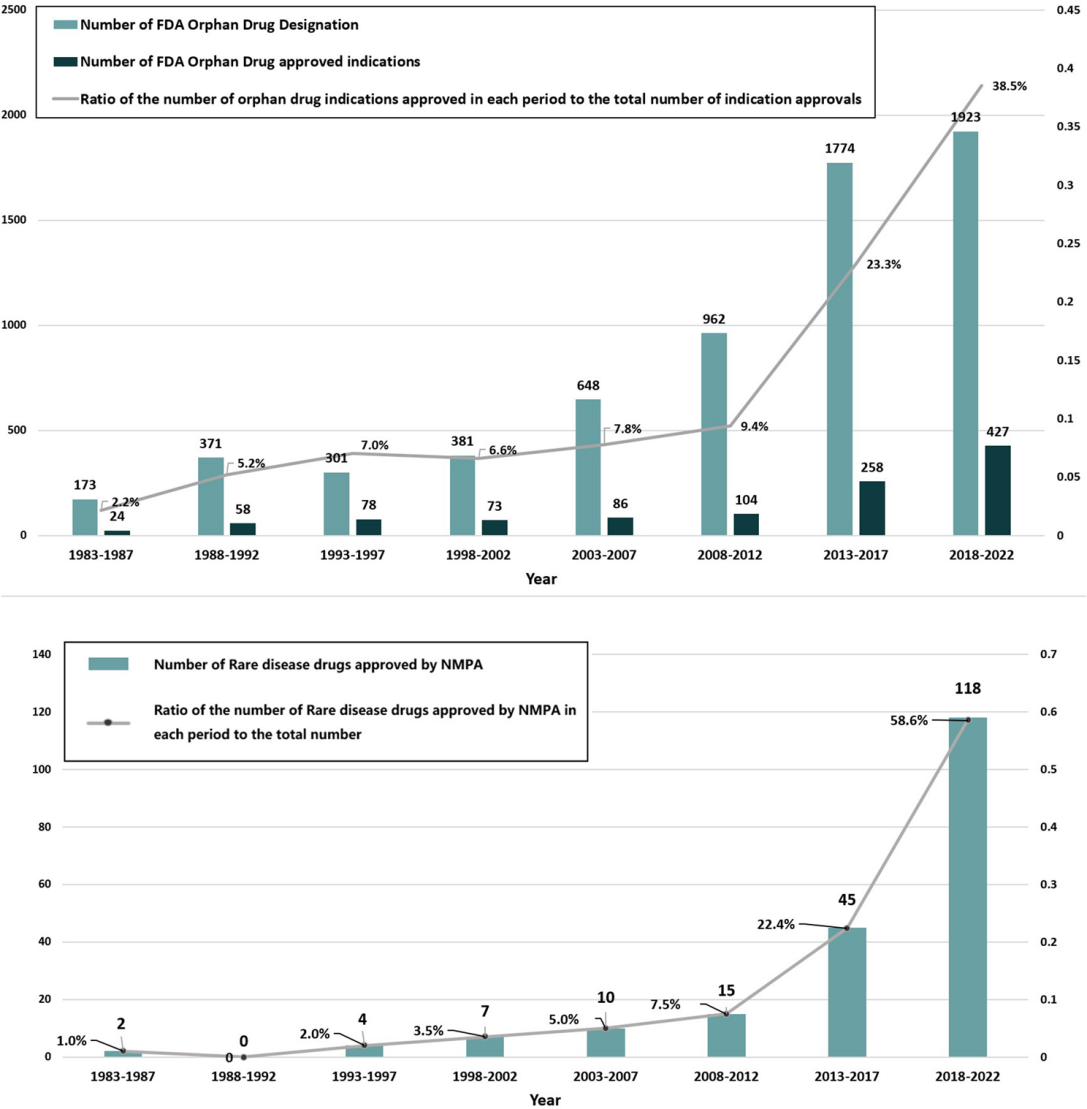
Overview of the number of orphan drugs approved by the FDA and Number of FDA-approved orphan drugs which are approved by NMPA

Of the 693 orphan drugs approved in the USA, 201 (29.0%) were also approved in China. In terms of specific dosage forms and specifications, 425 out of 1,228 (34.6%) available in the USA gained approval in China. Figure 1 illustrates the distribution of rare disease drugs approved in China and their proportion relative to all USA-approved orphan drugs.

### Lag

#### Approval lag time between FDA and NMPA

**Fig. 2:**
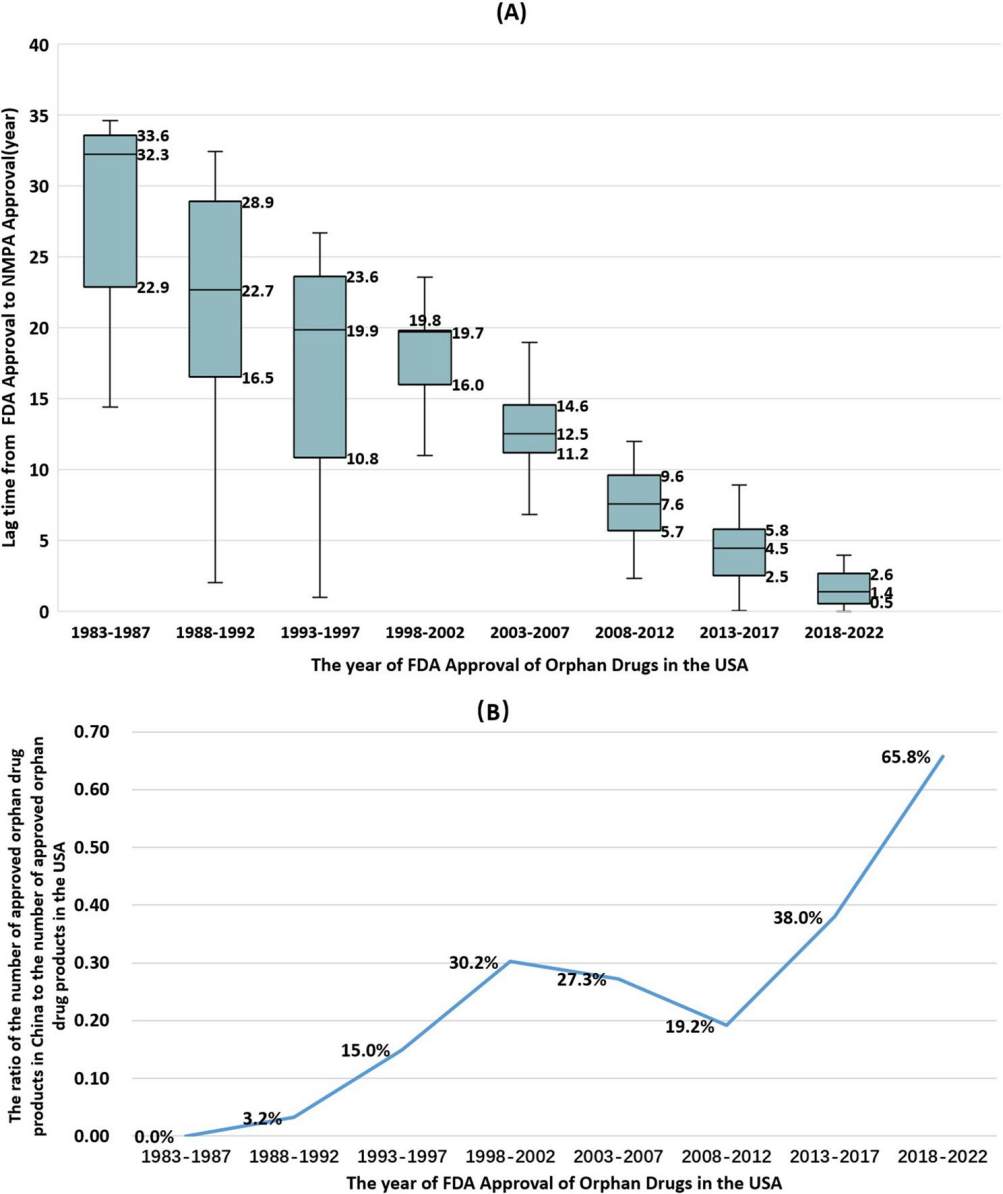
Lag time to market and ratio of orphan drugs in China and the USA. (**A**) Lag time from FDA orphan drug market approval to NMPA market approval. (The horizontal line in the box plot represents the median, the shaded area represents the range of quartiles, and the whisker area represents the maximum and minimum values).(**B**) Changes in the ratio of the number of approved orphan drug products in China to the number of approved orphan drug products in the USA in 1983–2022

The approval lag was calculated based on the difference in the initial approval dates in the USA and China for the 425 dosage forms/specifications that were approved in both countries. Over the past four decades (1983–2022), the disparity in orphan drug approval timelines between China and the USA has narrowed significantly, the pace of orphan drug approval in China has accelerated markedly. The lag time from FDA orphan drug approval to NMPA approval is shown in Fig. 2A. The median approval lag time was 32.3 years(IQR: 22.9–33.6)from 1983 to 1987. In contrast, the shortest median lag time occurred between 2018 and 2022, at approximately 1.4 years (IQR: 0.5–2.6)—a reduction of 94.2% that reflects the success of China’s ongoing drug regulatory reforms.

Figure 2B shows the ratio of orphan drug products approved in China to those approved in the USA over time. This ratio has increased steadily since 2008, further demonstrating the progress in China’s orphan drug approval process.

### Characteristics

#### Utilization of expedited programs for FDA-Approved orphan drugs (1983–2022)

**Fig. 3:**
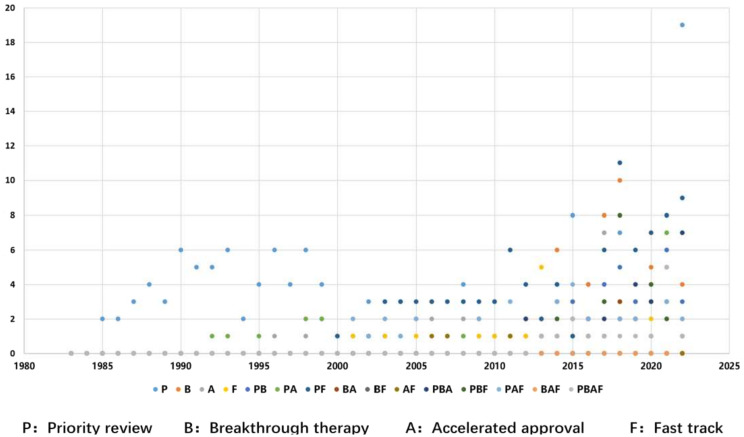
Four expedited programs among orphan drugs approved by the FDA from 1983 to 2022

To accelerate the development and review of drugs for serious conditions, the FDA established four expedited programs: Priority Review (P, 1992), Breakthrough Therapy (B, 2012), Accelerated Approval (A, 1992), and Fast Track (F, 1997). Figure 3 illustrates the application of these programs for orphan drugs approved between 1983 and 2022. The use of expedited programs has increased substantially since 2013. Of the 617 drug indications that received Priority Review during the 40-year period, 58.0% (358/617) were granted to orphan drugs in the recent decade alone (2013–2022), underscoring a growing reliance on these mechanisms for rare disease therapies.

Since 2005, China’s National Medical Products Administration (NMPA) has introduced a series of regulatory policies to accelerate drug review and approval [20]. These comprise four expedited pathways, summarized in Table 1:

**Table 1:** Comparison of NMPA’s accelerated procedures and FDA’s expedited programs for drugs in the treatment of serious diseases

	Categorization	Qualification criteria	Creation time
NMPA	Breakthrough therapy	Innovative or improved new drugs that are used to prevent or treat serious life-threatening diseases or diseases that seriously affect the quality of life and for which there is no effective means of prevention or treatment, or for which there is sufficient evidence of a clear clinical advantage over existing means of treatment, etc., during the period of clinical trials of drugs	2020
	Conditional approval	(a) Drugs for the treatment of serious life-threatening diseases for which there is no effective means of treatment, where data from clinical trials of the drugs are available to confirm the efficacy of the drugs and predict their clinical value; (b) Drugs urgently needed for public health, where data from clinical trials of the drugs are available to show the efficacy of the drugs and predict their clinical value; (c) Vaccines urgently needed to cope with major public health emergencies, or other vaccines recognized as urgently needed by the National Health Commission, where the benefits of the vaccines have been assessed to be greater than the risks.	2017
	Priority review	(a) Urgent clinical shortage of drugs, innovative and improved drugs to prevent and treat major infectious diseases and rare diseases and other diseases; (b) In line with the physiological characteristics of children’s new varieties of medicines for children, dosage forms and specifications; (c) Disease prevention, control of vaccines and innovative vaccines in urgent need; (d) Included in the breakthrough therapeutic drug program; (e) In line with the approval of the conditions attached to the drugs; (f) The State Drug Administration stipulates that the other priority situations for review and approval.	2015
	Special approval	In the event of the threat of public health emergencies and public health emergencies after the occurrence of public health emergencies, the State Drug Administration may decide in accordance with the law on public health emergencies required for emergency prevention and treatment of drugs.	2005
FDA	Breakthrough Therapy Designation	Breakthrough Therapy designation is a process designed to expedite the development and review of drugs that are intended to treat a serious condition and preliminary clinical evidence indicates that the drug may demonstrate substantial improvement over available therapy on a clinically significant endpoint(s).	2012
	Accelerated approval	A positive therapeutic effect that is clinically meaningful in the context of a given disease is known as “clinical benefit”. Mindful of the fact that it may take an extended period of time to measure a drug’s intended clinical benefit, Accelerated Approval allowed drugs for serious conditions that filled an unmet medical need to be approved based on a surrogate endpoint. Using a surrogate endpoint enabled the FDA to approve these drugs faster.	1992
	Priority Review Designation	A Priority Review designation means FDA’s goal is to take action on an application within 6 months (compared to 10 months under standard review). It will direct overall attention and resources to the evaluation of applications for drugs that, if approved, would be significant improvements in the safety or effectiveness of the treatment, diagnosis, or prevention of serious conditions when compared to standard applications.	1992
	Fast Track Designation	Fast track is a process designed to facilitate the development, and expedite the review of drugs to treat serious conditions and fill an unmet medical need. The purpose is to get important new drugs to the patient earlier. Fast Track addresses a broad range of serious conditions.	1997

Special approval (2005): Applies to innovative or improved drugs for serious or life-threatening conditions with no effective treatment, or those demonstrating clear clinical advantages during clinical trials.

Priority review (2015): Grants expedited review to drugs with obvious clinical value may apply for the application of the priority review procedure;

Conditional approval (2017): Allows earlier approval based on promising clinical data for critical illnesses or urgent public health needs so that they can be used as soon as possible for patients with critical illnesses or urgent public health needs who cannot wait any longer. Pharmaceuticals granted conditional approval are required to complete confirmatory studies to comprehensively validate their safety, efficacy, and clinical benefit. These confirmatory studies must be concluded within the validity period of the drug registration certificate, which is typically five years.

Breakthrough therapy (2020): Designated during Phase I or II clinical trials (typically before Phase III begins) to encourage the development of drugs with clear clinical advantages [21].

**Fig. 4:**
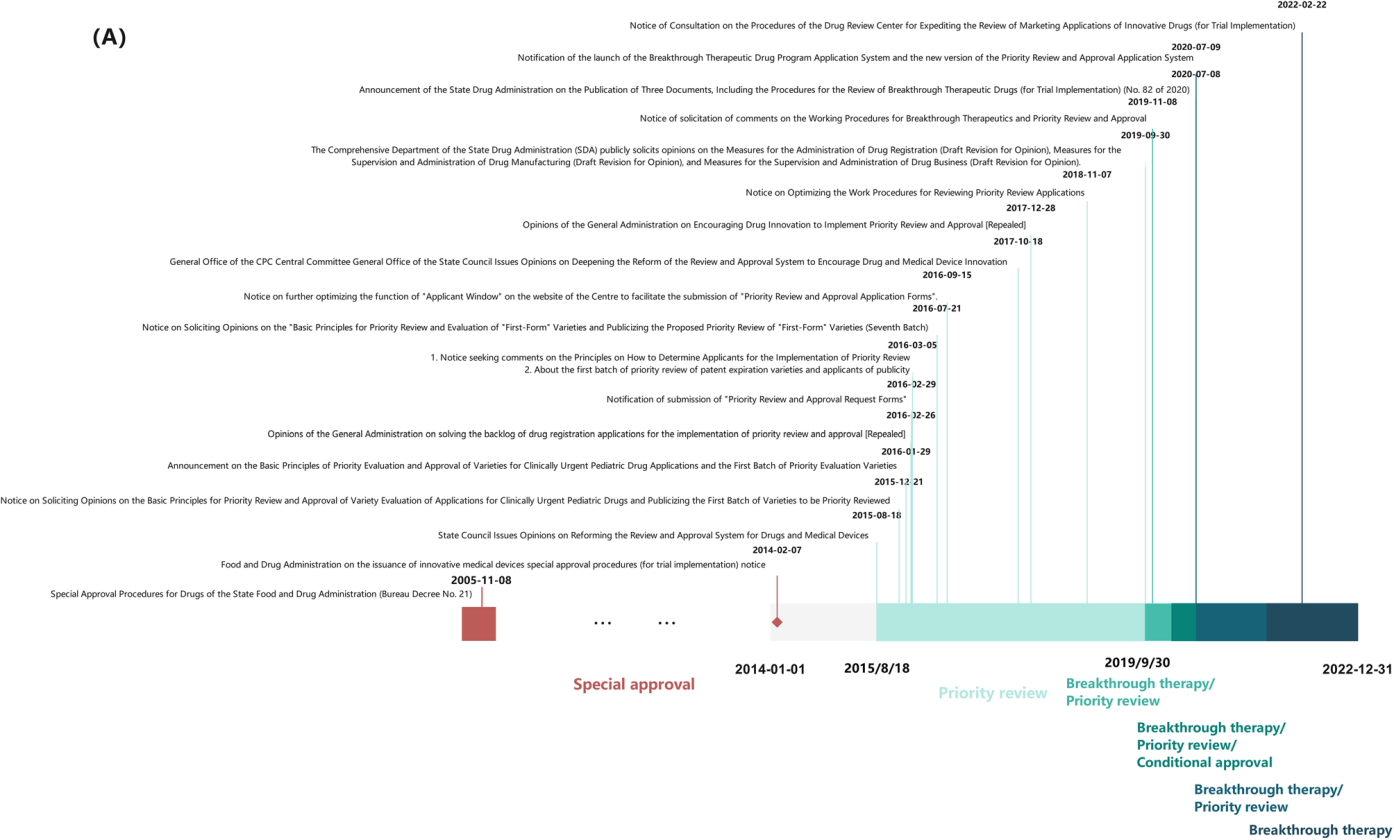
Laws and regulations related to accelerated drug review and approval processes in China

The chronological rollout of these pathways is summarized in Fig. 4. Their implementation has significantly accelerated drug approval in China [22], including for rare disease drugs, especially over the past nine years (2014–2022). This correlation is evident when comparing the increasing number of NMPA approvals (Fig. 1) with policy milestones (Fig. 4). The growing emphasis on rare disease drugs is further demonstrated by the number granted expedited status: in 2016, there were 12 rare disease drugs included in the four special pathways, 18 in 2017, 32 in 2018, 22 in 2019, 31 in 2020, 18 in 2021, and 17 in 2022. In our analysis of USA orphan drug policies, regulations, and guidelines from 1983 to 2022, annual data revealed distinct chronological trends in designation-to-approval timelines. Between 1983 and 2005, the interval fluctuated between 4 and 10 years. However, a consistent decline occurred from 2006 onward: decreasing from 8.3 years (2006) to 0.3 years (2022), with intermediate values of 6.4, 7.3, 5.5, 6.4, 5.0, 4.3, 4.3, 1.0, 3.2, 3.5, 3.2, 2.0, 1.8, 0.9, and 0.5 years.

**Fig. 5:**
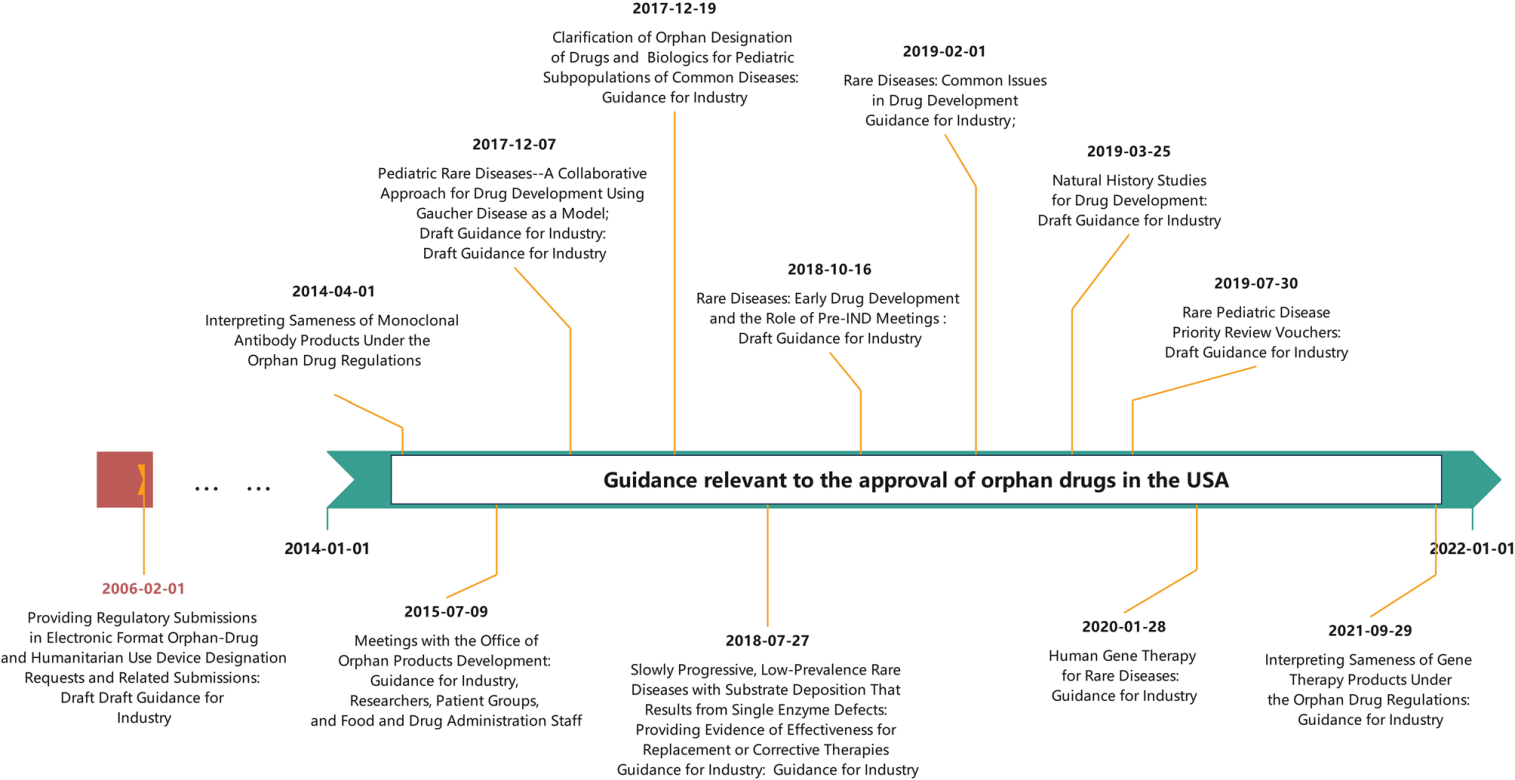
Guidance relevant to the approval of orphan drugs in the USA

This improvement in the USA was supported by key regulatory updates, such as the 2006 guidance Providing Regulatory Submissions in Electronic Format for Orphan-Drug and Humanitarian Use Device Designation Requests, which enhanced the efficiency of the review process and facilitated orphan drug approvals (Fig. 5).

## Discussion

### The significant increase in NMPA approvals for rare disease drugs

Over the past four decades (1983–2022), the number of FDA approved orphan drugs also approved in China has been increasing and the time lag between FDA and Chinese approval has been decreasing. This progress coincides with the development and refinement of multiple expedited regulatory pathways in China. To date, only three published studies have focused explicitly on drug approval lags in China compared to other countries [23–25]. This study uses the rate at which drug regulators assess and approve rare disease drugs as a metric of regulatory capacity and efficiency. Clarifying the characteristics of these approval lags and identifying their key drivers may provide insights for the Chinese government to implement more targeted measures to narrow the drug access gap [23]. This study uses the rate at which drug regulators assess and approve rare disease drugs as a metric of regulatory capacity and efficiency. Clarifying the characteristics of these approval lags and identifying their key drivers may provide insights for the Chinese government to implement more targeted measures to narrow the drug access gap [26]. Guided by the “Healthy China” strategy, the country has increasingly prioritized the prevention and treatment of rare diseases. A series of policy documents have been issued to promote drug R&D and improve treatment access and healthcare security for rare disease patients. Key measures include:

(i) Establishing an efficient review and regulatory process by clearing the backlog of overdue applications [27].
(ii) Implementing expedited review channels that can reduce approval delays by approximately 30 months [20, 23].
(iii) Lifting restrictions on the early development of imported drugs and increasing participation in global drug research [28, 29].
(iv) Granting eligibility for all imported rare disease drugs to apply for priority review, with a statutory review time limit of 10 days [14].

Furthermore, this wave of reforms led to a comprehensive revision of the Drug Administration Law in 2019 and the rewriting of numerous important regulations [30]. A milestone was reached when the International Council for Harmonisation (ICH) General Assembly accepted the NMPA as a regulatory member, signifying China’s emergence as a global player in pharmaceutical regulation.

Additional initiatives include the 2019 establishment of the National Rare Disease Diagnosis and Treatment Collaborative Network by China’s National Health Commission. From 2018 to 2020, the NMPA published three batches of a “List of Urgently Needed Overseas New Drugs,” which included 73 drugs (33 for rare diseases). This list focused on new drugs—already approved in the USA, EU, or Japan but not yet in China—for treating serious conditions with unmet medical needs. Drugs on this list were prioritized for review and approval. In 2021, the Guiding Principles for Clinical Research and Development of Rare Disease Drugs were released to further improve the efficiency of clinical development. These concerted efforts partly explain why China’s drug approval times for rare diseases have recently approached those of the USA A related study by our team showed an upward trend in the availability of rare disease drugs in China; by 2020, median hospital-level availability reached 41.1%, with a 16.5% increase in the number of highly available drugs. Furthermore, 64 out of 74 orphan drugs were considered affordable for urban and rural residents covered by the National Basic Medical Insurance, representing an increase of 14.1% [31].

Despite differences in political systems, economic development, and culture between China and the United States, the USA FDA’s orphan drug designation mechanism offers valuable insights for China. A clear and standardized definition of orphan drugs is fundamental to the designation process [32–34]. An interview-based study in China revealed that 78.6% of respondents (representatives from patient organizations) supported incentives for rare disease research, and 71.4% recommended enacting orphan drug legislation [35]. Therefore, building on studies of orphan drug designation in the USA and other countries [36], we recommend that China establish formal definitions for rare diseases and orphan drugs based on epidemiological, clinical effectiveness, and economic criteria [32, 37], while also considering national conditions. Such definitions would provide a solid foundation for implementing subsequent incentives for orphan drug research and development [38, 39] and offer a legal basis for improving treatment and protection for patients with rare diseases in China.

A limitation of this study arises because the NMPA does not disclose when drug developers submit applications, only the final approval date. This lack of transparency regarding the review timeline prevents a more granular analysis of the approval process and represents an important area for future investigation.

### Factors driving the significant increase in FDA orphan drug designations and approvals

The total number of FDA orphan drug approvals has increased significantly over the past 40 years. Orphan drugs account for a significant proportion of new molecular entities approved by the FDA in the last 5 years [40]. This is the result of a combination of factors [4, 40–44]: (1) policy and guidelines factors: The Orphan Drug Act (ODA), enacted in January 1983 and subsequently amended in 1984, 1985, 1988, and 2017, established a foundational incentive framework. This was bolstered by the Rare Diseases Act of 2002, which provided additional research funding and legal support. A major catalyst was the FDA’s Orphan Drug Modernization Plan (June 2017), which committed to processing all backlogged designation applications within 90 days and ensuring all new applications receive a response within 90 days. This policy dramatically increased industry engagement. Current incentives include a 25% tax credit for clinical trial costs (formerly 50%), waiver of New Drug Application (NDA) and Biologics License Application (BLA) fees, eligibility for research grants, access to expedited approval pathways, exemptions from certain clinical data requirements, and seven years of market exclusivity post-approval. (2) Technological factors: Approximately 80% of rare diseases have a genetic origin. Breakthroughs in genomics, genetic sequencing, molecular biology, and biotechnology have enabled more precise drug targeting and significantly enhanced the R&D capabilities of pharmaceutical companies. Consequently, there has been a marked increase in approvals for biologics, drugs treating rare cancers, and targeted therapies. (3) Commercial incentives, including premium pricing and guaranteed market exclusivity, deliver high returns to manufacturers and strongly stimulate orphan drug development [28]. One study indicated that companies with orphan drug approvals achieve a 9.6% higher return on investment compared to their peers [45]. The *Evaluate Pharma Orphan Drug Report *2019 confirmed that orphan drugs command substantially higher prices in the USA market. However, these high prices and the market monopolies created by exclusivity—which can stifle competition—remain topics of intense debate and controversy [46–50].

## Conclusions

By analyzing the approval lag for rare disease drugs between the USA and China, this study reveals a clear trend: from 1983 to 2022, a growing number of these drugs have been approved in both countries, with the approval lag in China consistently decreasing. This indicates a degree of synchronization in global drug development and underscores China’s remarkable progress in catching up with international approval timelines.

The rapid increase in approvals over the past decade reflects the guiding role of China’s regulatory reforms and catalog-driven policy remain in a relatively early, though highly dynamic, phase of development.

Over the past 40 years, China has prioritized accelerating drug approval processes, implementing a range of policies to achieve this goal. Significant attention has been dedicated specifically to orphan drugs. The reform of China’s drug regulatory system has yielded substantial results in this field. This research provides clear evidence of breakthroughs in the review and approval of rare disease drugs in China, demonstrating the powerful impact of the ongoing restructuring and reform of the country’s regulatory ecosystem. These advances are expected to stimulate further development of rare disease treatments both in China and worldwide.

## Supplementary information

Below is the link to the electronic supplementary material.

Supplementary Material 1

Supplementary Material 2

Supplementary Material 3

Supplementary Material 4

## Data Availability

All data generated or analysed during this study are included in this published article [and its supplementary information files].
